# Supplementary material for: Optical opening of the blood-brain barrier for targeted and ultra-sparse viral infection of cells in mouse cortex
Source: Cell Rep Methods. 2023 Jun 2;3(6):100489. doi: 10.1016/j.crmeth.2023.100489 (PMC10326348; doi:10.1016/j.crmeth.2023.100489)

Cell Reports Methods, Volume *3*

Supplemental information

Optical opening of the blood-brain barrier for targeted and ultra-sparse viral infection of cells in mouse cortex

Patrick Reeson, Roobina Boghozian, Ana Paula Cota, and Craig E. Brown

Supplementary Information

Figure S1. Comparing the spatial extent of cre-mediated labelling of cells following intracortical micro-injection of AAV.hSyn.cre versus rupture of a single capillary, related to Figure 1. Confocal images from a coronal brain section show cre dependent expression of tdtomato 3 weeks after AAV injection with a glass micropipette (A; 0.4uL, ~1.3×10^{10} GCs) or after rupture of a capillary (B; i.v. injection of 6.92×10^{12} GCs/kg). Scale bar = 200µm.

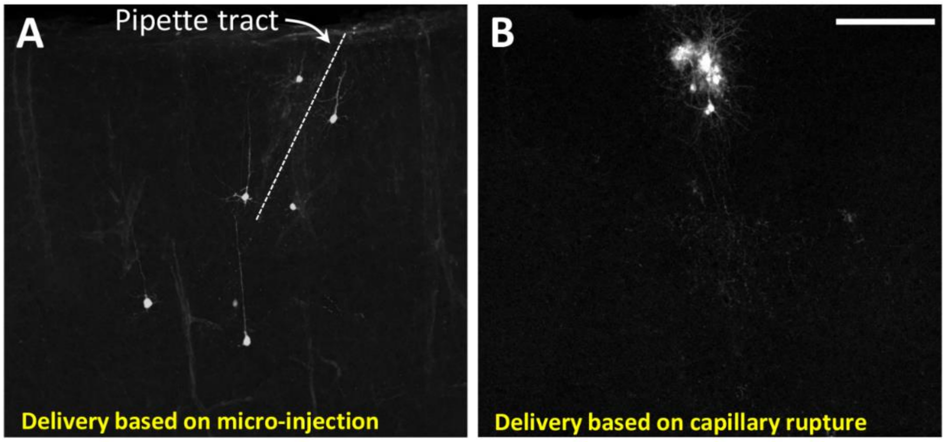

Figure S2. Time-dependent changes in microglial responses to capillary rupture, related to Figure 2 and 5. A.) Longitudinal *in vivo* imaging of microglia in male Cx3cr1$^{gfp/wt}$ mice before and up to 28 days after rupture of a single capillary. Note the rapid accumulation of microglia processes around the bleed site and delayed recruitment of cells 1 day after injury. The capillary is retained and regains blood flow while microglial reactivity subsides over 14 days after rupture. B.) Representative examples from 4 additional mice at day 0, 1, 7 and 14. Scale bars = 20μm.

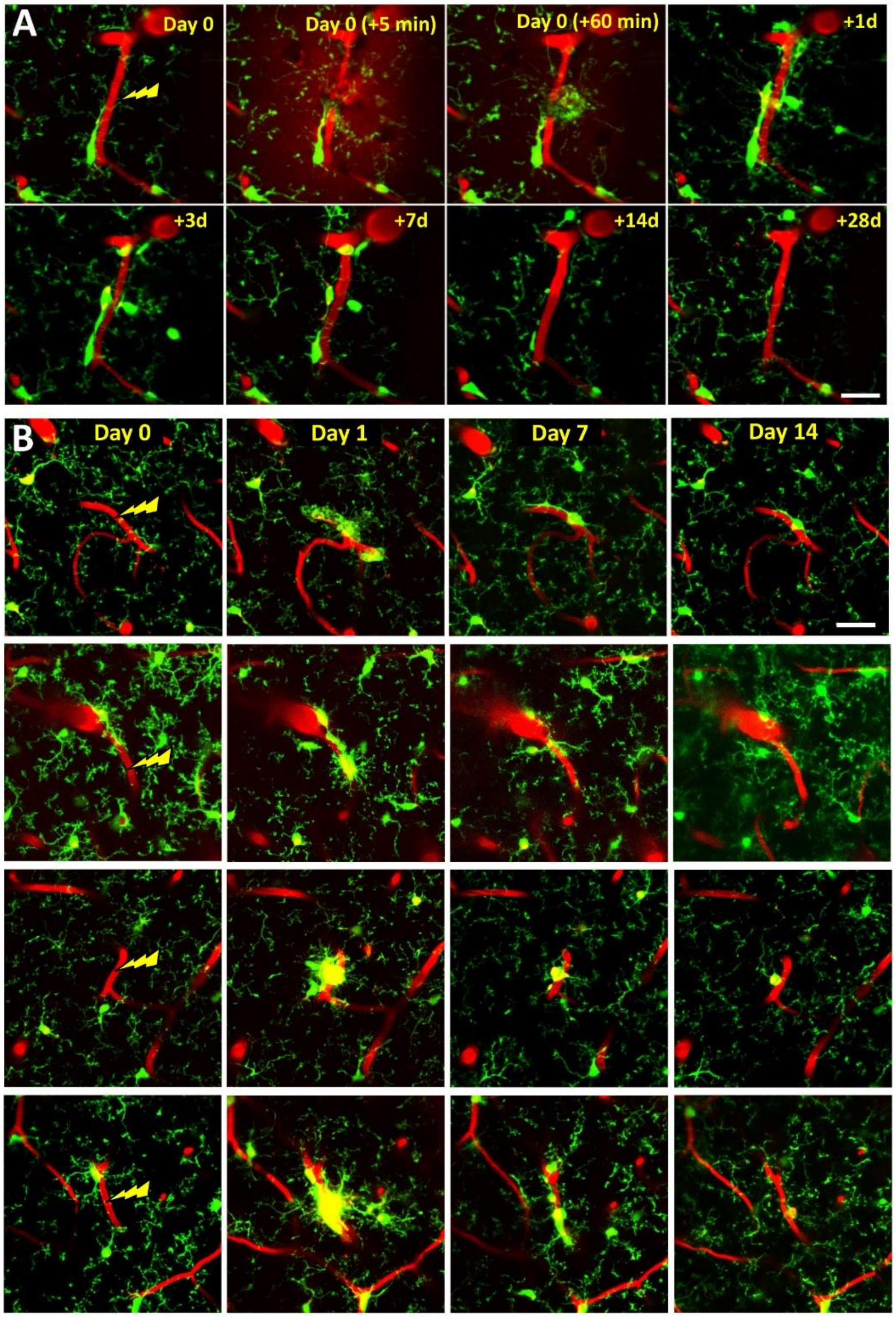

Supplement: Document S1. Figures S1 and S2 [file mmc1.pdf]
