# Supplementary material for: Optical opening of the blood-brain barrier for targeted and ultra-sparse viral infection of cells in mouse cortex
Source: Cell Rep Methods. 2023 Jun 2;3(6):100489. doi: 10.1016/j.crmeth.2023.100489 (PMC10326348; doi:10.1016/j.crmeth.2023.100489)

Cell Reports Methods

Article

Optical opening of the blood-brain barrier for targeted and ultra-sparse viral infection of cells in mouse cortex

Graphical abstract

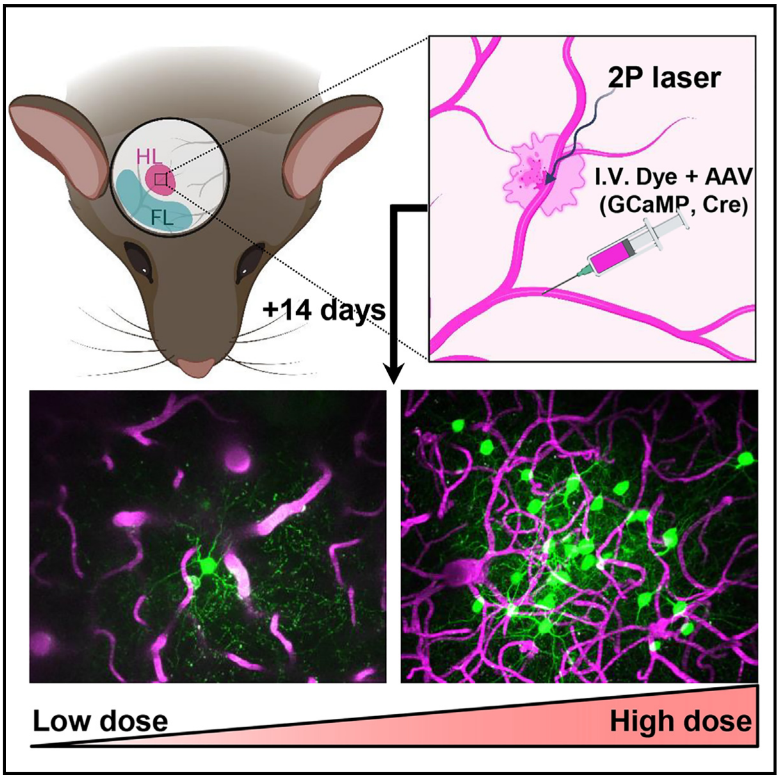

Highlights

- Spatially targeted and ultra-sparse AAV transduction of cells through cranial window
- Very limited tissue damage associated with optical AAV delivery method
- Transduction density can be titrated with AAV dose and diameter of vessels targeted
- Method can deliver cre and constitutive AAVs for GCaMP, ChR2, tdTomato expression

Authors

Patrick Reeson, Roobina Boghozian, Ana Paula Cota, Craig E. Brown

Correspondence

brownc@uvic.ca

In brief

Conventional methods for delivery of AAVs for *in vivo* imaging can be damaging and imprecise. Reeson et al. show that one can direct AAV expression to cortical cells in a targeted, minimally invasive, and titratable manner by optically perforating blood vessels through the cranial imaging window.

Reeson et al., 2023, Cell Reports Methods *3*, 100489
June 26, 2023 © 2023 The Author(s).
https://doi.org/10.1016/j.crmeth.2023.100489

CellPress

Cell Reports Methods

CellPress
OPEN ACCESS

Article

Optical opening of the blood-brain barrier for targeted and ultra-sparse viral infection of cells in mouse cortex

Patrick Reeson,[1] Roobina Boghozian,[1] Ana Paula Cota,[1] and Craig E. Brown[1,2,3,*]
[1]Division of Medical Sciences, University of Victoria, Victoria, BC V8P 5C2, Canada
[2]Department of Psychiatry, University of British Columbia, Vancouver, BC, Canada
[3]Lead contact
*Correspondence: brownc@uvic.ca
https://doi.org/10.1016/j.crmeth.2023.100489

MOTIVATION One lingering problem for the many labs that use multi-photon microscopy to image or activate AAV-transduced cells is the delivery method. Traditional approaches are either damaging, lack spatial precision, or require specialized equipment or reagents. Therefore, we developed a simple approach whereby the imaging laser can be used to precisely deliver AAVs for transducing cortical cells in a titratable, ultra-sparse, and minimally invasive manner.

SUMMARY

Adeno-associated viruses (AAVs) are used in a wide array of experimental situations for driving expression of biosensors, recombinases, and opto-/chemo-genetic actuators in the brain. However, conventional approaches for minimally invasive, spatially precise, and ultra-sparse AAV-mediated transduction of cells during imaging experiments have remained a significant challenge. Here, we show that intravenous injection of commercially available AAVs at different doses, combined with laser-based perforation of cortical capillaries through a cranial widow, allows for ultra-sparse, titratable, and micron-level precision for delivery of viral vectors with relatively little inflammation or tissue damage. Further, we show the utility of this approach for eliciting sparse expression of GCaMP6, channelrhodopsin, or fluorescent reporters in neurons and astrocytes within specific functional domains in normal and stroke-damaged cortex. This technique represents a facile approach for targeted delivery of viral vectors that should assist in the study of cell types and circuits in the cortex.

INTRODUCTION

The rapid expansion of genetic and optical tools for monitoring and manipulating cells in the rodent brain has redefined how neuroscientists study brain structure and function. For example, neuroscientists often employ adeno-associated viruses (AAVs) to genetically modify brain cells to make them amenable for imaging.[1] However the delivery of these vectors, which typically relies on blood-brain barrier (BBB)-permeable AAVs or direct micro-injection, can be spatially imprecise, technically challenging with risk of infection and hemorrhage, and, worst of all, damaging to the same regions one intends to image.

There are several new tools available that enable minimally invasive expression of AAVs in the rodent brain. The recent development of blood-brain barrier-permeable AAVs with cell type-specific promoters provides a new alternative for widespread expression of specific proteins in the brain without the need for micro-injections.[2,3] For spatially precise expression of proteins within a particular cell, Yao et al.[4] created light-inducible recombinases that can be activated *in vivo* with single- and two-photon light sources. Adding another layer of specificity, there are also new viral toolkits that incorporate Boolean logic to precisely control gene expression within defined cell types.[5–8] While revolutionary, the application of these methods has been slowed by the fact that delivery of the payload (e.g., light-sensitive cre-recombinase) involves micro-injection or the aforementioned BBB-permeable AAVs, which can yield capricious expression in some mouse strains or avoid certain cells *in vivo*.[9] Another recent approach that has generated tremendous excitement is the use of focused ultrasound (FUS) to remotely and transiently disrupt the BBB with micro-bubbles in order to deliver AAVs.[10–13] The benefits of this method is that one can non-invasively deliver AAVs to any brain region of interest. However, some limitations are the need for potentially expensive equipment to implement FUS, the inability to control the extent of transfection on a micro-meter scale, and the unavoidable sterile inflammation found within the volume of tissue targeted by FUS. While all these different approaches have enormous potential for

This is an open access article under the CC BY license (http://creativecommons.org/licenses/by/4.0/).

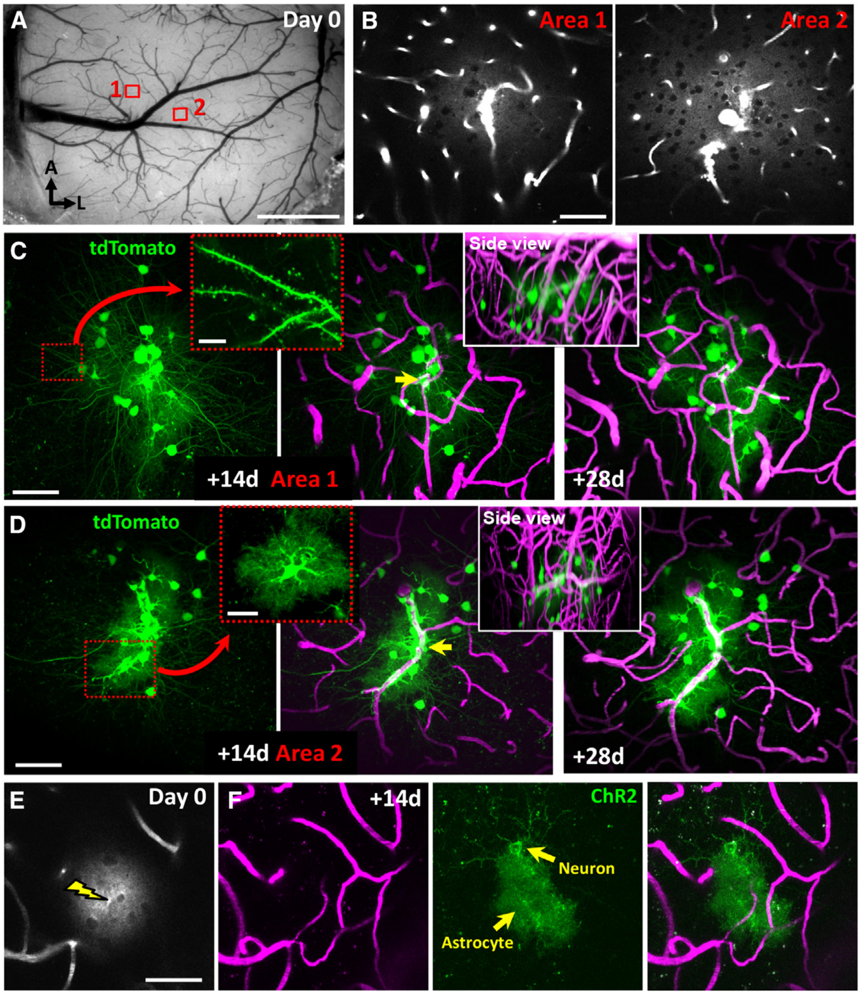

Figure 1. Targeted delivery of AAVs and sparse expression of cre-recombinase-dependent fluorescent proteins in cortical neurons

(A) Bright-field image of the cortical surface in an Ai9 reporter mouse implanted with a cranial window and intravenously injected with AAV1.hSyn.-cre. Note the two areas where a single capillary was ruptured in each.

(B) Two-photon images showing extravasation of fluorescently labeled blood plasma in areas 1 and 2 immediately after rupturing a capillary.

(C and D) *In vivo* maximal intensity z-projection images taken 14 and 28 days (left and right, respectively) after vessel rupture in areas 1 and 2, showing the cre-dependent expression of tdTomato in nearby cells (green) and vasculature labeled with fluorescent dye (magenta). Note the bright tdTomato signal that allows visualization of fine dendritic structure or peri-vascular astrocytes (see insets in C and D, respectively). Insets show y-z or "side-view" image projections at 28 days post-transfection.

(E) Image showing extravasation of plasma dye immediately after vessel rupture in an Ai32 mouse.

(F) *In vivo* maximal intensity z-projection images taken 14 days after vessel rupture revealing the Cre-dependent expression of EYFP-labeled ChR2(H134R) in an Ai32 mouse.

Scale bars: in (A), 1 mm, in (B)–(E) 50 μm, and insets in (C) and (D), 10 μm.

minimally invasive, spatially targeted delivery of AAVs or expression of cre-dependent proteins, they are not ideally suited or sufficiently simple for all experimental applications.

To address this need, we have optimized a simple yet effective and titratable method to achieve targeted, ultra-sparse AAV transfection of cortical neurons and astrocytes in the cerebral cortex. This facile approach involves the intravenous administration of commercially available AAVs followed by optically puncturing single capillaries with the same femtosecond laser used to image cells *in vivo.*[14] Since the dose of AAVs or the number or size of capillaries targeted can be titrated, the extent of cellular transfection (tdTomato reporter, GCaMP, ChR2) can be manipulated. Furthermore, the extent of inflammation and putative tissue damage is extremely limited compared with traditional micro-injection procedures, thereby allowing one to image cells at the target site with minimal optical distortion, which invariably accompanies tissue damage (e.g., edema).

RESULTS

Conventional delivery of AAVs using micro-injection is technically challenging and inevitably leads to considerable tissue damage associated with the micro-pipettes. As an alternative, we considered the possibility that intravenous injection of an AAV followed by laser-based perforation of a capillary could provide precise and minimally invasive delivery. Our rationale was based in part on the fact that sparse cre-recombinase-dependent reporter expression in the brain can be achieved with direct micro-injection of a very dilute solution of virus (e.g., 1:20,000 dilution; see Figure S1). Given the blood volume of an adult mouse is approximately 1.5–2.5 mL, it was reasonable to think that a comparable dilution could be attained with an intravenous injection of a high-titer AAV. Therefore, we intravenously injected AAV1.hSyn.cre.WPRE.hGh (Addgene #105553, 6.92 × 10^{12} GC/kg) diluted in 2.5%–5% fluorescein isothiocyanate (FITC) dextran (70 kDa; Sigma-Aldrich #46945) into adult mice implanted with a cranial window (Figure 1A) that conditionally expresses the fluorescent reporter tdTomato (Ai9, JAX# 007909). To precisely target cells within a specific cortical region, we optically perforated a single capillary (circular region of interest [ROI] 3–4 μm in diameter was placed at the edge of the capillary) between 50 and 250 μm below the cortical surface with our high-power femtosecond laser. For rupturing vessels deeper in the cortex, increased laser power and higher concentration of plasma dye will be needed. However, one must proceed with caution given that the point spread function will broaden with deeper ablations, thus potentially compromising focality. Capillaries between 3 and 7 μm in diameter that were clearly in focus

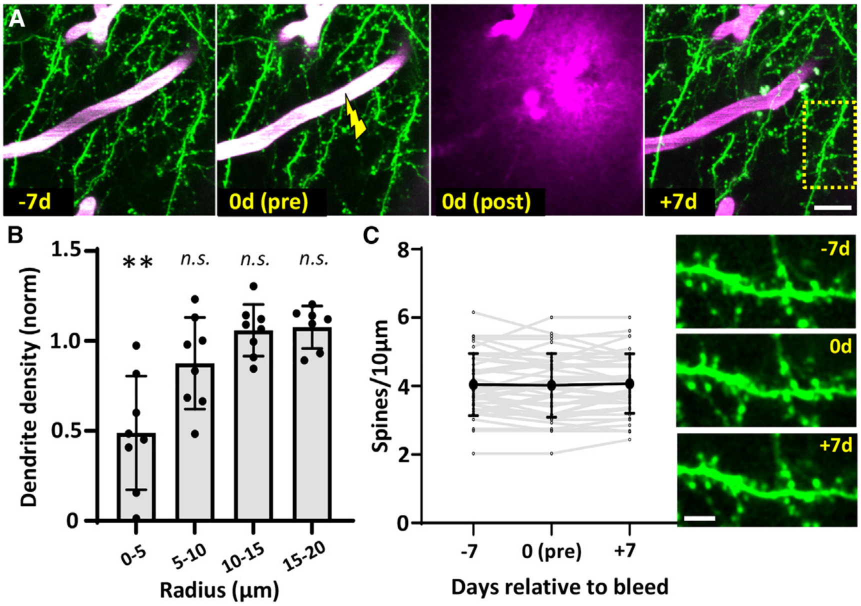

Figure 2. Effects of capillary perforation on local dendritic structure

(A) *In vivo* two-photon images show cortical dendrites 7 days before and after rupture of a capillary at day 0.

(B) The density of nearby dendrites was significantly reduced within a 5 μm radius from the rupture site but not at greater distances (7 ruptures from 4 female mice).

(C) Dendritic spine density from branches within 40 μm radius of the rupture did not change significantly (one-way ANOVA $F_{(2,117)} = 0.03$, $p = 0.97$; 40 branches sampled in 4 female mice). Representative image projections showing dendritic spines before and after rupture.

Statistics were based on one-sample t test (B) and one-way ANOVA (C). Data are presented as mean ± standard error. **$p < 0.01$. Scale bars: in (A), 10 μm, and in (C), 5 μm.

and ran parallel to the imaging plane (an at least 10 μm segment) were selected for perforation. Puncture of a capillary was easily confirmed by the appearance of an extravascular dye fluorescence plume surrounding the rupture (Figure 1B). Reimaging the same region 2 and 4 weeks later revealed sparsely labeled neurons and astrocytes adjacent to the ruptured capillary (Figures 1C and 1D). Our success rate in achieving cre-dependent tdTomato expression was 95.6% (22/23 ruptures in 4 male mice). We should note that since we ruptured capillaries in multiple cortical regions (≥500 μm from each other) over the span of 60 min after AAV injection, we did not find any time-dependent decrement in successful AAV-mediated cell labeling. To prove this approach could be applied to other cre-dependent strains, we injected AAV1.hSyn.cre (intravenous [i.v]. 6.92 × 10^{12} GC/kg) into mice that conditionally express YFP-tagged channelrhodopsin-2 (ChR2; Ai32, JAX #024109). Doing so led to ChR2 expression in neurons and astrocytes next to the ruptured capillary (Figures 1E and 1F; 100% success rate from 11 ruptures in 2 male mice; Ai32, JAX #024109). We should also note that in 4 mice, we attempted to transfect cells with AAV in new regions 6 weeks after a previous injection of AAV. However, these attempts in the second round were unsuccessful in all 4 mice, likely due to the production of AAV-neutralizing antibodies, which has been reported in other studies with different AAVs.[15,16]

An important question to address is the extent to which the rupture of a capillary induces local tissue damage. Previous studies from our lab and others[17,18] have shown that inflammation from microglia peaks within 1–4 days after micro-bleed and then subsides by 2 weeks recovery (see Figure S2). However, to what extent neurons and fine synaptic structure are affected is not well established. To address this, we longitudinally imaged the local dendritic structure before and after capillary rupture. Our analysis indicated that the density of cortical dendrites was reduced by 50% within a 5 μm radius from the rupture, whereas density beyond 5 μm was not significantly affected (Figures 2A and 2B; one-sample t tests, 0–5 μm $t_{(7)} = 4.57$, $p = 0.002$; for radii >5 μm all $p > 0.05$). This reduction within 5 μm could reflect actual tissue damage or perhaps tissue displacement with the extravasation of a few red blood cells. With respect to dendritic spines, we did not see any change in spine density after capillary rupture (Figure 2C). Thus, the present results in tandem with previous work showing that sensory-evoked calcium responses recover within 24 h after induction of micro-bleed,[19] suggest that tissue damage, if any, is very minimal.

Next, we wanted to determine if other AAVs (such as constitutive ones) could be delivered and express their payload without the need for a cre-dependent mouse strain. Intravenous injection of AAV1.CAG.tdTomato (5.06 × 10^{12} GC/kg; Addgene #59462) followed by capillary perforation induced tdTomato expression in neurons and astrocytes 2–3 weeks later (Figure 3A; 100% success in 15 ruptures from 2 male mice). Although we could detect labeled cells in each experiment, the brightness of tdTomato expression was considerably lower than the cre-dependent expression of tdTomato in the Ai9 reporter strain (using the same excitation wavelength and laser power). In our next set of experiments, we tested a comparable dose of AAV1.hSyn.GCamP6s.WPRE.SV40 (6.67 × 10^{12} GC/kg, Addgene #100843). In this case, we functionally mapped the forelimb and hindlimb somatosensory cortex using intrinsic signal optical imaging and targeted single capillaries in these regions (Figures 3B and 3C). Two to three weeks later, we could detect GCamP6s-expressing neurons and astrocytes near the site of rupture (88% success from 25 ruptures in 4 male mice; Figure 3C). To determine if these cells were viable and active, we imaged neuronal calcium transients in response to 1 s vibrotactile stimulation of the contralateral limb (Figure 3D). Analysis of 15 GCaMP6s-expressing neurons from 3 mice indicated that 7/15 neurons in the somatosensory cortex were reliably responsive to tactile stimulation (average peak dF/Fo = 45.4% ± 32.5%),

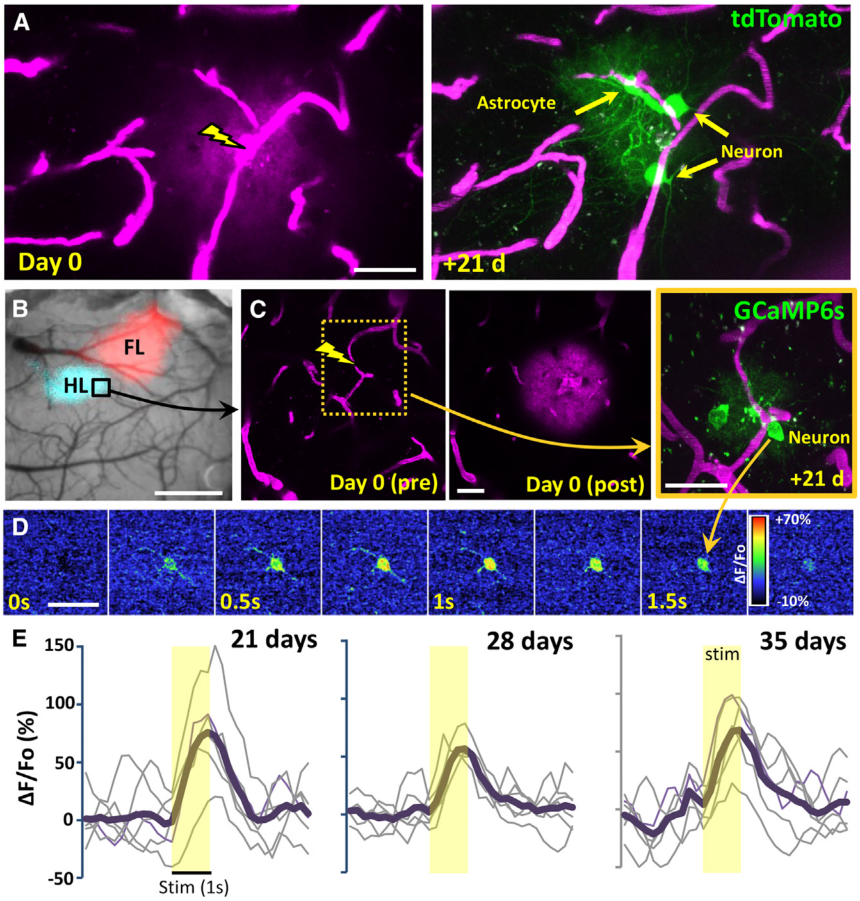

Figure 3. Targeted delivery and expression of constitutive (non-cre-dependent) AAVs to cortical neurons

(A) Two-photon maximum intensity projection images showing rupture of a cortical capillary after intravenous injection of constitutive AAV (AAV1.CAG.tdTomato) and resultant expression of tdTomato in cortical astrocytes and neurons 3 weeks later.

(B) Bright-field image showing the cortical surface with intrinsic optical signal-derived maps of the forelimb (FL) and hindlimb (HL) primary somatosensory cortex superimposed on top.

(C) Two-photon images show a capillary immediately before and after rupture on day 0, as well as resulting GCaMP6s expression in nearby cells 21 days later.

(D) Color montage illustrates the time course of hindlimb-evoked neuronal calcium responses (average of 6 stimulation trials, expressed as % ΔF/Fo).

(E) Individual and averaged (6 trials, thick black line) calcium responses (from neuron shown in C and D) following 1 s vibrotactile stimulation of the contralateral hindlimb, collected at 21, 28, and 35 days after vessel rupture.

Scale bars: in (A), (C), and (D), 30 μm, and in (B), 1 mm.

which fits with previous imaging data.[20,21] As shown in Figure 3E, tactile stimulation evoked calcium transients in the same neuron over multiple weeks. Importantly the long-term sensory responsiveness of these neurons indicates that the cells in the immediate vicinity of the perforated capillary remain functional and appear to suffer no ill effects from the transient rupture. Collectively, these experiments indicate that constitutive AAVs (ie. non-cre-recombinase dependent) can be delivered and expressed in the mouse cortex using our approach.

For imaging and understanding the wiring diagram of cortical neurons at different scales, it would be helpful to titer AAV-mediated expression in targeted regions. Therefore, we i.v. injected 3 different doses of AAV1.hSyn.cre.WPRE.hGh in Ai9 tdTomato reporter mice. Comparison of reporter expression 2 weeks after capillary perforation indicated a dose-dependent increase in cells expressing the tdTomato reporter (Figures 4A and 4B; Kruskal-Wallis test, main effect of dose: $p < 0.0001$). At the lowest dose (1×: 1.73 × 10^{12} GC/kg), tdTomato-labeled cells were found at 24 of 26 sites (92.3% success) with a median of 2 cells per site (Figure 4B). With higher doses, the success rate increased (95.6% and 100% for 4× and 8×, respectively), as did the median number of labeled cells per site (left panel in Figure 4B; 6 and 17 cells/site for 4× and 8× doses, respectively). By examining the morphology of labeled cells (right panel in Figure 4B), the proportion of neuronal vs. astroglial cells labeled was generally not significantly different except for the medium dose. Next, we examined the proximity of labeled cells to the rupture site. Our analysis shows that on average, neurons were located 60.62 μm away, whereas astrocytes were significantly closer at an average distance of 38.93 μm (Figure 4C; unpaired t test, $p = 0.04$). And finally, since capillaries can vary in diameter (from 3 to 7 μm), we plotted the number of labeled cells per site as a function of lumen diameter (Figure 4D). Linear regression analysis indicated a significant relationship ($R^2 = 0.137$, $p = 0.014$), suggesting that rupturing larger capillaries tends to label more AAV-infected cells. In summary, these results show that AAV-mediated transfection of cortical cells leads to spatially localized expression that can be titered by dose and the size of the vessel perforated.

While there are many possible applications for this method, we highlight one example focused on cortical plasticity following stroke. Our lab and several others[21] have used longitudinal two-photon imaging through a cranial window to describe structural and functional changes to cortical circuits in the days and weeks that follow an ischemic stroke in the forelimb somatosensory cortex.[20,22] An obvious, yet until now very difficult experiment, would be to functionally identify the part of the forelimb cortex that emerges weeks after stroke (so called "reorganized" or "reemergent" cortical representation) and use AAVs to image and/or map their connections. Using a conventional micro-injection approach would be problematic because it lacks precision and would cause further damage to peri-infarct tissues, which are already vulnerable to insults, especially the vasculature. As shown in Figure 5, we identified

CellPress
OPEN ACCESS

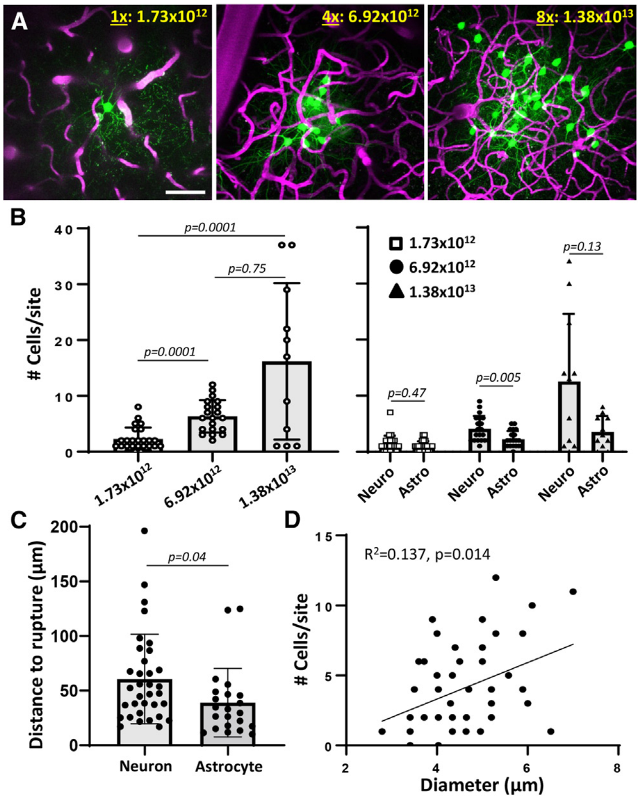

Figure 4. Titered expression of cre-dependent AAVs in cortical neurons allow for ultra-sparse labeling applications

(A) *In vivo* maximum intensity z-projection images collected from an Ai9 tdTomato reporter mice 2 weeks after intravenous injection of AAV1.hSyn.cre and targeted rupture of a capillary. Note the increase in tdTomato-expressing cells from low to higher doses of AAV (1×, 4×, and 8× with 3, 4, and 3 male mice respectively; AAV dose expressed GC/kg).

(B) Left: bar graphs show a significant effect of AAV dose (GC/kg) on the number of cells labeled per site (Kruskal-Wallis statistic: 20.11, $p < 0.0001$). Right: the number of neurons or astrocytes per site as a function of AAV dose.

(C) Graph shows that neurons were located slightly, but significantly, further from the site of rupture than astrocytes for mice injected with the lowest dose of AAV.

(D) Linear regression showing relationship between the diameter of ruptured capillaries and the number of labeled cells.

Statistics based on Kruskal-Wallis statistic with post-hoc Dunn's (B: left) or Mann-Whitney (B: right), unpaired t test (C), and linear regression (D). Data are presented as mean ± standard error. Scale bar: in (A), 50 µm.

the forelimb primary somatosensory cortex before and after photothrombotic stroke using intrinsic optical signal imaging (Figure 5A) and then targeted AAV-mediated tdTomato expression to peri-infarct cells (Figures 5B and 5C). Importantly, we did not see overt signs of tissue damage in the form of generalized vessel loss or abnormal permeability of plasma dye across the BBB in subsequent imaging sessions (Figure 5D). The fact that ruptured capillaries recover and recanalize after 2 weeks (see examples in Figures 1E, 2A, 3A, 3B, and 5D) agrees with previous work from our lab[17] and also correlates with the resolution of microglia-related inflammation around the rupture site (Figure S2).

DISCUSSION

Here, we have validated a simple, minimally invasive approach for sparse and spatially targeted AAV expression in the mouse cortex. This method leverages a common tool in neuroscience laboratories, the two-photon microscope, which most labs interested in optical reporters and actuators already have and use. We exploited the fundamental advantage of multi-photon excitation, which is spatially restricted to a focal point of excitation,[23,24] to perform targeted perforations of cortical capillaries. When combined with AAVs injected into the bloodstream, this transient rupture allows an extremely small quantity of viral particles into the cortical parenchyma and transfection of only a few adjacent neurons and astrocytes. We further demonstrate that this method is an effective tool for targeted and limited expression of different AAVs and genetic payloads. We also have shown that by varying the dose of AAV and the diameter of vessels targeted, one can titrate the level of expression from just one or two cells to several dozen. Lastly, we demonstrate a practical application of this methodology, driving AAV-dependent expression of tdTomato in a sparse set of surviving peri-infarct neurons. The advantages of this methodology are its ability to precisely deliver AAVs with micron-level precision and sparsely label neurons at a density of one's choosing without risking damage associated with direct micro-injection.

Direct micro-injections remain the most common method for gene transduction in the brain using viral vectors, especially when the goal is focal uptake of an AAV and expression of the transgene. This method is effective, relatively simple, and cost effective but remains limited for selective targeting. Titrating viral loads, and thus expression, with glass pipettes requires either excessive dilutions or extremely small volumes. Neither of these strategies circumvent the inherent lack of precision of inserting a ~5- to 200-µm-wide glass pipette (from tip to further up the bevel) into the cortex. Additionally the insertion inevitably leaves a path of damage and AAV backflow up the insertion track and less precise expression (Figure S1). The method described in the present study is not the only approach to improve upon the traditional method of AAV delivery by insertion of a glass pipette. For example, recent studies have proven that FUS combined with i.v.-injected micro-bubbles can deliver systemically administered AAVs to any brain region.[11,25] While this approach could be a transformative step for clinical application of gene therapy, BBB disruption and gene transduction occur over a relatively large volume (~0.125–1 mm^3), and therefore it is not suitable for micron-level precision of AAV delivery and longitudinal imaging of sparsely labeled cells. Another major innovation was the development of light-inducible cre recombinases.[4] These constructs work along similar principles: the transgene of interest is widely expressed, usually by micro-injection or i.v. infusion of BBB-permeable AAV-PHP,[26] followed by focal application of light to activate cre recombinase. A significant advantage of this method is that it could allow for ultra-sparse and circuit-specific manipulations by chaining different intersectional criteria, such as cell-specific cre expression, cell-specific transgenes, and the spatial/temporal application of light. While this method holds tremendous potential, the ability to target cells within a very specific region is dependent and conceivably limited by

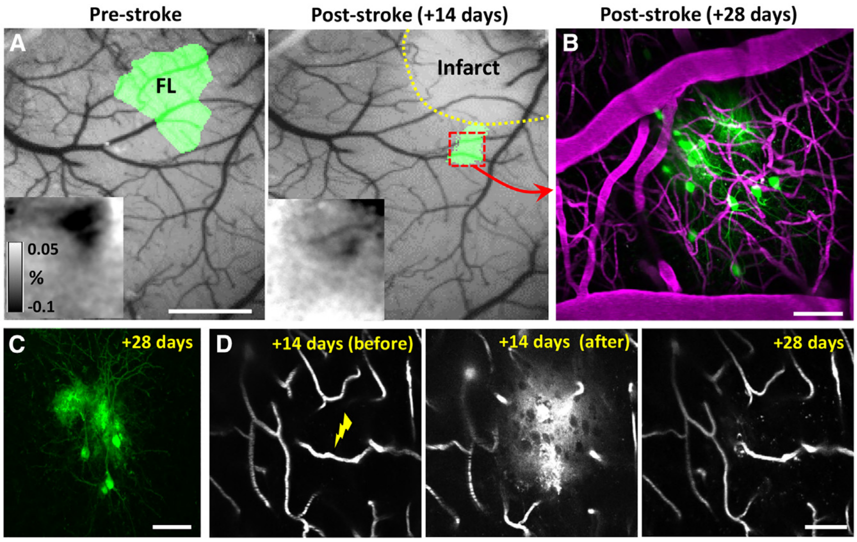

Figure 5. Application of the method for examining structural plasticity in cortical neurons within functionally altered somatosensory maps after stroke

(A) Bright-field images of the cortical surface overlaid with intrinsic optical signal maps of the forelimb somatosensory cortex before and 14 days after focal ischemic stroke. Insets show intrinsic optical signal reflectance maps (% dR/Ro) generated from stimulating the contralateral forelimb.

(B) *In vivo* maximum intensity projection image showing tdTomato-expressing cells within the remaining portion of forelimb map 2 weeks after induction of vessel rupture and intravenous injection of AAV1.hSyn.cre (6.92×10^{12} GC/kg; see middle panel in D).

(C) Post-mortem confocal image (from coronal brain section) showing tdTomato-labeled cells displayed in (B).

(D) Two-photon images showing the targeted capillary immediately before (left) or after (middle) rupture. Imaging the same capillary 2 weeks later (right, +28 days) shows that it was preserved and regained blood flow.

Scale bars: in (A), 1 mm, and in (B)–(D), 50 μm.

how well the existing AAV-PHPs can deliver the light-inducible recombinase. For example, it is known that efficiency of AAV-PHP transduction is variable and dependent on factors such as mouse strain[2,9] and exhibits a tropism for certain neurons (e.g., pyramidal neurons in cortical layers 2/3 and 5), although cell specificity has been improving. To obviate this issue, direct micro-injection of AAV-PHP has been used,[4] although for the reasons previously stated, the invasive effects of direct injection are less than ideal for *in vivo* imaging. Since the viruses used in our study lack cellular specificity, we can envision combining the optical viral delivery approach of the present study with new viral toolkits that provide “Boolean logic” to drive gene expression within specific cell types.[5–8]

Any new approach is only as useful as its best applications. Here, we used this approach to address a challenge we have considered for years: how to non-invasively label a sparse set of surviving peri-infarct neurons within small fragments of the stroke-affected somatosensory cortex. While we have simply shown proof of concept here, our approach allows us to target surviving neurons and trace their projections, whose locations are notoriously difficult to predict after stroke. Moreover, we can restrict AAV-delivered opto- or chemo-genetic actuators to neurons in these surviving regions. Therefore, one could precisely manipulate their function/activity and potential role in stroke recovery with simple, imprecise light sources (e.g., surface LEDs) or systemic administration of chemogenetic ligands.[20,22] The spatial resolution of this method is also advantageous for investigating finely organized topological maps, such as targeting functional subdomains within a single whisker barrel[27] or retinotopic/feature-specific micro-domains in the visual cortex.[28,29] Several recent groundbreaking studies have used retrograde viral constructs to trace presynaptic inputs to a single post-synaptic neuron infected by single-cell delivery with a glass pipette.[28–30] This method is extremely challenging and potentially damages incident axons to the target neuron as the pipette is moved into a juxtasomal position. Therefore, an optical approach for delivery of viral constructs to single neurons could significantly improve the success rate of these experiments and lower the expertise needed to implement them. Lastly, the method presented here could be useful for precise and very focal delivery of drugs into the brain. This might be of benefit to experimental neuroscientists who would want to achieve focal drug delivery with minimal tissue damage.

Limitations of the study

While we believe the approach described in the present article will be useful to the multi-photon imaging community, there are important limitations that should be considered. Firstly, our approach is not completely non-invasive since we still have to optically puncture a micro-vessel for AAV delivery. However, consistent with previous work,[19] we show that cells in the immediate vicinity of the ruptured capillary display preserved activity patterns, namely sensory-evoked responsiveness over weeks time. Consistent with this, dendritic branches and spines were mostly unaffected by the rupture except within a 5 μm radius. Further, punctured vessels regained blood flow and local inflammatory microglial responses subsided within 2 weeks. A second limitation is that our approach does not allow one to pick a specific cell or cell type for viral transfection, therefore expression is somewhat random within the zone of transfection. A third limitation is we only tested AAV serotype 1 and in mice with a C57 background. Thus, we cannot guarantee that this approach will work for all AAVs or animals tested. Case in point, we noted lower tdTomato reporter expression associated with the constitutive AAV compared with cre-dependent tdTomato expression, thus for some AAVs or applications, our approach may not provide optimal protein expression. However, we should note that capillary perforation delivery of AAV.syn.cre worked very well in both Ai9 and Ai32 reporter strains, suggesting this is a robust delivery method, at least when used with cre-dependent mouse

CellPress
OPEN ACCESS

strains. Furthermore, the delivery of these viruses is likely based on passive diffusion through the ruptured vessel for at least 30 min after rupture (but not more than 24 h)[17,31] rather than an active receptor-based transport (e.g., LY6A receptor needed for AAV-PHP), which can limit AAV delivery in certain mouse strains. Another limitation is that systemic administration of AAV and uptake of virus throughout the body raise the possibility of organ toxicity. Although our mice did not display signs of morbidity with any AAV dose tested (pain- or sickness-related behaviors) for at least 4–6 weeks after injection, future refinements could incorporate intranasal delivery of AAVs, which leads to viral expression in the brain but with significantly lower biodistribution in peripheral organs.[25] We should also note that our attempts to transfect new cells several weeks after a first round of AAV-mediated transfection were unsuccessful, likely due to the production of AAV-neutralizing antibodies.[15,16] And finally, the present method is limited by the need for a cranial window and the inherent depth limitations of two-photon imaging, particularly given the high laser powers required for vessel perforation.[17,31] While light scattering in tissue is a fundamental limit of any optical method, these concerns can be managed by surgical expertise, choice of cranial window (open vs. thinned skull),[27] and emerging deep-tissue imaging methods such as three-photon imaging and gradient-index (GRIN) lenses. Presumably, any laboratory that is currently using two-photon microscopy for *in vivo* imaging could easily apply this technique to their study with minimal cost and no need for additional equipment.

STAR★METHODS

Detailed methods are provided in the online version of this paper and include the following:

- KEY RESOURCES TABLE
- RESOURCE AVAILABILITY
  - Lead contact
  - Materials availability
  - Data and code availability
- EXPERIMENTAL MODEL AND STUDY PARTICIPANT DETAILS
- METHOD DETAILS
  - Cranial window surgery
  - Intrinsic optical signal (IOS) imaging
  - *In vivo* two-photon imaging and targeted delivery of AAVs to cortical regions
- QUANTIFICATION AND STATISTICAL ANALYSIS

SUPPLEMENTAL INFORMATION

Supplemental information can be found online at https://doi.org/10.1016/j.crmeth.2023.100489.

ACKNOWLEDGMENTS

We are grateful to Angie Hentze and Taimei Yang for managing the mouse colony. We thank Stephanie Taylor and Eslam Mehina for microglia images. Work was supported by operating, salary, and equipment grants to C.E.B. from the Canadian Institutes of Health Research (CIHR), the Heart and Stroke Foundation (HSF), and the Natural Sciences and Engineering Research Council (NSERC).

AUTHOR CONTRIBUTIONS

C.E.B. conceived of the study. P.R. and C.E.B. co-wrote the manuscript. C.E.B. performed experiments and collected data. C.E.B. and A.P.C. performed data analysis. R.B. and P.R. performed mouse surgeries.

DECLARATION OF INTERESTS

The authors declare no competing interests.

Received: November 11, 2022
Revised: March 20, 2023
Accepted: May 10, 2023
Published: June 2, 2023

REFERENCES

1. Nectow, A.R., and Nestler, E.J. (2020). Viral tools for neuroscience. Nat. Rev. Neurosci. *21*, 669–681. https://doi.org/10.1038/s41583-020-00382-z.
2. Hordeaux, J., Yuan, Y., Clark, P.M., Wang, Q., Martino, R.A., Sims, J.J., Bell, P., Raymond, A., Stanford, W.L., and Wilson, J.M. (2019). The GPI-linked protein LY6A drives AAV-PHP.B transport across the blood-brain barrier. Mol. Ther. *27*, 912–921. https://doi.org/10.1016/j.ymthe.2019.02.013.
3. Liu, D., Zhu, M., Zhang, Y., and Diao, Y. (2021). Crossing the blood-brain barrier with AAV vectors. Metab. Brain Dis. *36*, 45–52. https://doi.org/10.1007/s11011-020-00630-2.
4. Yao, S., Yuan, P., Ouellette, B., Zhou, T., Mortrud, M., Balaram, P., Chatterjee, S., Wang, Y., Daigle, T.L., Tasic, B., et al. (2020). RecV recombinase system for in vivo targeted optogenomic modifications of single cells or cell populations. Nat. Methods *17*, 422–429. https://doi.org/10.1038/s41592-020-0774-3.
5. Fenno, L.E., Mattis, J., Ramakrishnan, C., Hyun, M., Lee, S.Y., He, M., Tucciarone, J., Selimbeyoglu, A., Berndt, A., Grosenick, L., et al. (2014). Targeting cells with single vectors using multiple-feature Boolean logic. Nat. Methods *11*, 763–772. https://doi.org/10.1038/nmeth.2996.
6. Fenno, L.E., Ramakrishnan, C., Kim, Y.S., Evans, K.E., Lo, M., Vesuna, S., Inoue, M., Cheung, K.Y.M., Yuen, E., Pichamoorthy, N., et al. (2020). Comprehensive dual- and triple-feature intersectional single-vector delivery of diverse functional payloads to cells of behaving mammals. Neuron *107*, 836–853.e11. https://doi.org/10.1016/j.neuron.2020.06.003.
7. Ubina, T., Vahedi-Hunter, T., Agnew-Svoboda, W., Wong, W., Gupta, A., Santhakumar, V., and Riccomagno, M.M. (2021). ExBoX – a simple Boolean exclusion strategy to drive expression in neurons. J. Cell Sci. *134*, jcs257212. https://doi.org/10.1242/jcs.257212.
8. Pouchelon, G., Vergara, J., McMahon, J., Gorissen, B.L., Lin, J.D., Vormstein-Schneider, D., Niehaus, J.L., Burbridge, T.J., Wester, J.C., Sherer, M., et al. (2022). A versatile viral toolkit for functional discovery in the nervous system. Cell Rep. Methods *2*, 100225. https://doi.org/10.1016/j.crmeth.2022.100225.
9. Huang, Q., Chan, K.Y., Tobey, I.G., Chan, Y.A., Poterba, T., Boutros, C.L., Balazs, A.B., Daneman, R., Bloom, J.M., Seed, C., et al. (2019). Delivering genes across the blood-brain barrier: LY6A, a novel cellular receptor for AAV-PHP.B capsids. PLoS One *14*, e0225206. https://doi.org/10.1371/journal.pone.0225206.
10. Kofoed, R.H., Heinen, S., Silburt, J., Dubey, S., Dibia, C.L., Maes, M., Simpson, E.M., Hynynen, K., and Aubert, I. (2021). Transgene distribution and immune response after ultrasound delivery of rAAV9 and PHP.B to the brain in a mouse model of amyloidosis. Mol. Ther. Methods Clin. Dev. *23*, 390–405. https://doi.org/10.1016/j.omtm.2021.10.001.
11. Kofoed, R.H., Dibia, C.L., Noseworthy, K., Xhima, K., Vacaresse, N., Hynynen, K., and Aubert, I. (2022). Efficacy of gene delivery to the brain using AAV and ultrasound depends on serotypes and brain areas. J. Control. Release *351*, 667–680. https://doi.org/10.1016/j.jconrel.2022.09.048.

12. Noroozian, Z., Xhima, K., Huang, Y., Kaspar, B.K., Kügler, S., Hynynen, K., and Aubert, I. (2019). MRI-guided focused ultrasound for targeted delivery of rAAV to the brain. Methods Mol. Biol. *1950*, 177–197. https://doi.org/10.1007/978-1-4939-9139-6_10.

13. Weber-Adrian, D., Kofoed, R.H., Silburt, J., Noroozian, Z., Shah, K., Burgess, A., Rideout, S., Kügler, S., Hynynen, K., and Aubert, I. (2021). Systemic AAV6-synapsin-GFP administration results in lower liver biodistribution, compared to AAV1&2 and AAV9, with neuronal expression following ultrasound-mediated brain delivery. Sci. Rep. *11*, 1934. https://doi.org/10.1038/s41598-021-81046-5.

14. Rosidi, N.L., Zhou, J., Pattanaik, S., Wang, P., Jin, W., Brophy, M., Olbricht, W.L., Nishimura, N., and Schaffer, C.B. (2011). Cortical microhemorrhages cause local inflammation but do not trigger widespread dendrite degeneration. PLoS One *6*, e26612. https://doi.org/10.1371/journal.pone.0026612.

15. Halbert, C.L., Rutledge, E.A., Allen, J.M., Russell, D.W., and Miller, A.D. (2000). Repeat transduction in the mouse lung by using adeno-associated virus vectors with different serotypes. J. Virol. *74*, 1524–1532. https://doi.org/10.1128/JVI.74.3.1524-1532.2000.

16. Rapti, K., Louis-Jeune, V., Kohlbrenner, E., Ishikawa, K., Ladage, D., Zolotukhin, S., Hajjar, R.J., and Weber, T. (2012). Neutralizing antibodies against AAV serotypes 1, 2, 6, and 9 in sera of commonly used animal models. Mol. Ther. *20*, 73–83. https://doi.org/10.1038/mt.2011.177.

17. Mehina, E.M.F., Taylor, S., Boghozian, R., White, E., Choi, S.E., Cheema, M.S., Korbelin, J., and Brown, C.E. (2021). Invasion of phagocytic Galectin 3 expressing macrophages in the diabetic brain disrupts vascular repair. Sci. Adv. *7*, eabg2712. https://doi.org/10.1126/sciadv.abg2712.

18. Ahn, S.J., Anrather, J., Nishimura, N., and Schaffer, C.B. (2018). Diverse inflammatory response after cerebral microbleeds includes coordinated microglial migration and proliferation. Stroke *49*, 1719–1726. https://doi.org/10.1161/STROKEAHA.117.020461.

19. Cianchetti, F.A., Kim, D.H., Dimiduk, S., Nishimura, N., and Schaffer, C.B. (2013). Stimulus-evoked calcium transients in somatosensory cortex are temporarily inhibited by a nearby microhemorrhage. PLoS One *8*, e65663. https://doi.org/10.1371/journal.pone.0065663.

20. Motaharinia, M., Gerrow, K., Boghozian, R., White, E., Choi, S.-E., Delaney, K.R., and Brown, C.E. (2021). Longitudinal functional imaging of VIP interneurons reveals sup-population specific effects of stroke that are rescued with chemogenetic therapy. Nat. Commun. *12*, 6112. https://doi.org/10.1038/s41467-021-26405-6.

21. Zeiger, W.A., Marosi, M., Saggi, S., Noble, N., Samad, I., and Portera-Cailliau, C. (2021). Barrel cortex plasticity after photothrombotic stroke involves potentiating responses of pre-existing circuits but not functional remapping to new circuits. Nat. Commun. *12*, 3972. https://doi.org/10.1038/s41467-021-24211-8.

22. Tennant, K.A., Taylor, S.L., White, E.R., and Brown, C.E. (2017). Optogenetic rewiring of thalamocortical circuits to restore function in the stroke injured brain. Nat. Commun. *8*, 15879. https://doi.org/10.1038/ncomms15879.

23. Denk, W., Strickler, J.H., and Webb, W.W. (1990). Two-photon laser scanning fluorescence microscopy. Science *248*, 73–76. https://doi.org/10.1126/science.2321027.

24. Denk, W., Delaney, K.R., Gelperin, A., Kleinfeld, D., Strowbridge, B.W., Tank, D.W., and Yuste, R. (1994). Anatomical and functional imaging of neurons using 2-photon laser scanning microscopy. J. Neurosci. Methods *54*, 151–162. https://doi.org/10.1016/0165-0270(94)90189-9.

25. Ye, D., Yuan, J., Yang, Y., Yue, Y., Hu, Z., Fadera, S., and Chen, H. (2022). Incisionless targeted adeno-associated viral vector delivery to the brain by focused ultrasound-mediated intranasal administration. EBioMedicine *84*, 104277. https://doi.org/10.1016/j.ebiom.2022.104277.

26. Chan, K.Y., Jang, M.J., Yoo, B.B., Greenbaum, A., Ravi, N., Wu, W.-L., Sánchez-Guardado, L., Lois, C., Mazmanian, S.K., Deverman, B.E., et al. (2017). Engineered AAVs for efficient noninvasive gene delivery to the central and peripheral nervous systems. Nat. Neurosci. *20*, 1172–1179. https://doi.org/10.1038/nn.4593.

27. Drew, P.J., Shih, A.Y., Driscoll, J.D., Knutsen, P.M., Blinder, P., Davalos, D., Akassoglou, K., Tsai, P.S., and Kleinfeld, D. (2010). Chronic optical access through a polished and reinforced thinned skull. Nat. Methods *7*, 981–984. https://doi.org/10.1038/nmeth.1530.

28. Rossi, L.F., Harris, K.D., and Carandini, M. (2020). Spatial connectivity matches direction selectivity in visual cortex. Nature *588*, 648–652. https://doi.org/10.1038/s41586-020-2894-4.

29. Wertz, A., Trenholm, S., Yonehara, K., Hillier, D., Raics, Z., Leinweber, M., Szalay, G., Ghanem, A., Keller, G., Rózsa, B., et al. (2015). PRESYNAPTIC NETWORKS. Single-cell-initiated monosynaptic tracing reveals layer-specific cortical network modules. Science *349*, 70–74. https://doi.org/10.1126/science.aab1687.

30. Keller, A.J., Roth, M.M., and Scanziani, M. (2020). Feedback generates a second receptive field in neurons of the visual cortex. Nature *582*, 545–549. https://doi.org/10.1038/s41586-020-2319-4.

31. Taylor, S., Mehina, E., White, E., Reeson, P., Yongblah, K., Doyle, K.P., and Brown, C.E. (2018). Suppressing interferon-γ stimulates microglial responses and repair of microbleeds in the diabetic brain. J. Neurosci. *38*, 8707–8722. https://doi.org/10.1523/JNEUROSCI.0734-18.2018.

32. Reeson, P., Choi, K., and Brown, C.E. (2018). VEGF signaling regulates the fate of obstructed capillaries in mouse cortex. Elife *7*, e33670. https://doi.org/10.7554/eLife.33670.1.

CellPress
OPEN ACCESS

STAR★METHODS

KEY RESOURCES TABLE

REAGENT or RESOURCE	SOURCE	IDENTIFIER
Bacterial and virus strains		
pENN.AAV1.hSyn.Cre.WPRE.hGH	Addgene	RRID:Addgene_105553
pAAV1-CAG-tdTomato (codon diversified)	Addgene	RRID:Addgene_59462
pENN.AAV1.CamKII.GCaMP6f.WPRE.SV40	Addgene	RRID:Addgene_100834
Chemicals, peptides, and recombinant proteins		
FITC-dextran 70kDa	Sigma-Aldrich	CAT: 46945
Experimental models: Organisms/strains		
Mouse: B6.Cg-*Gt(ROSA)26Sor*$^{tm9(CAG\text{-}tdTomato)Hze}$/J	The Jackson Laboratory	RRID:IMSR_JAX:007909
Mouse:C57BL/6-Tmem119em1(cre/ERT2)Gfng/J	The Jackson Laboratory	RRID:IMSR_JAX:031820
Mouse:B6.Cg-Gt(ROSA)26Sortm32 (CAG-COP4*H134R/EYFP)Hze/J	The Jackson Laboratory	RRID:IMSR_JAX:024109
Mouse:B6.129P2(Cg)-Cx3cr1tm1Litt/J	The Jackson Laboratory	RRID:IMSR_JAX:005582
Mouse: B6.Cg-Tg(Thy1-YFP)HJrs/J	The Jackson Laboratory	RRID:IMSR_JAX:003782
Software and algorithms		
ImageJ	https://imagej.net/	RRID:SCR_003070
Excel	Microsoft	RRID:SCR_016137
Graphpad Prism	Dotmatics	RRID:SCR_002798

RESOURCE AVAILABILITY

Lead contact

Further information and requests for resources and reagents should be directed to and will be Fulfilled by the lead contact, Craig E. Brown (brownc@uvic.ca).

Materials availability

This study did not generate new unique reagents.

Data and code availability

- All data reported in this paper will be shared by the lead contact upon request
- This paper does not report original code.
- Any additional information required to reanalyze the data reported in this paper is available from the lead contact upon request.

EXPERIMENTAL MODEL AND STUDY PARTICIPANT DETAILS

Two to 12 month old mice on a C57BL/6J background were used in this study. All experiments were conducted in male mice (n = 25 males) except those involving the YFP-H line (n = 4 females). For experiments involving conditional cre-dependent expression of TdTomato, we utilized Ai9 reporter mice (B6.Cg-Gt(ROSA)26Sor$^{tm9(CAG\text{-}tdTomato)Hze\ 118/J}$, JAX# 007909) crossed with a microglia specific cre driver line (Tmem119$^{em1(cre/ERT2)Gfng\ 119/J}$, JAX# 031820). Cortical dendrites were imaged using thy1-YFP-H line of mice (B6.Cg-TgThy1-YFP HJrs/J; JAX#003782). In accordance with our ethical obligation to reduce animal numbers, some of these mice were re-used from another imaging study (unpublished study imaging microglia). We should note these mice were used as genetic controls in the previous study and had been given at least 8 weeks rest before being incorporated in the present study. Imaging of cre-dependent expression of eYFP tagged channelrhodopsin was achieved using Ai32 mice (B6.Cg-Gt(ROSA) 26Sort$^{m32(CAG-COP4*H134R/EYFP)Hze\ 124/J}$; JAX# 024109). And finally, constitutive eGFP expressing microglia were imaged using heterozygous Cx3cr1-eGFP mice (JAX # 005582). All mice were group housed (when possible) on a 12 h light/dark cycle in ventilated racks in a humidity (RH 40–55%) and temperature controlled room (21–23°C). For compassion of tittered expression of Cre AAV mice were randomly assigned to a viral titer group. Mice were provided food and water *ad libitum*. All experiments comply with the guidelines set by the Canadian Council on Animal Care and approved by the local university Animal Care Committee. Reporting of this work complies with ARRIVE guidelines.

METHOD DETAILS

Cranial window surgery

All mice imaged in the present study had a craniectomy based cranial window implanted over the right hemisphere. As previously described,[32] two to four month old mice were anesthetized with isoflurane (2% for induction and 1–1.3% for maintenance) mixed in medical air (flow rate: 0.7L/mL) and then fitted into a custom head fixing plate for surgery. Body temperature was maintained at 37°C with a feedback based heating system. After local injection of topical anesthetic, the scalp was cut and retracted. First, a 12 mm diameter titanium ring (used to hold the head during imaging, 7 mm inner diameter and 1.75 mm thick) was affixed to the skull with metabond adhesive. The skull was carefully thinned in a circular manner (~4mm diameter) with a dental drill until the bone became transparent. Fine foreceps were used to lift the skull flap off and ice-cold HEPES-buffered artificial cerebral spinal fluid (ACSF) was applied to the cortical surface. A 5 or 6mm circular coverslip was placed over the exposed brain and glued into place using cyanoacrylate glue and dental cement. Mice were allowed to recover on top of a heating blanket and then returned to their home cage.

Intrinsic optical signal (IOS) imaging

For IOS imaging, mice were lightly anesthetized with isoflurane (1% in medical air) and mounted on an upright Olympus microscope with body temperature maintained at 37°C. First, high contrast images of the surface vasculature were generated by illuminating the surface with white light and collecting reflected light through an ×2 objective (NA = 0.14) and a YFP emission filter. For each IOS imaging trial, we focused a red LED light (635nm, ~20 mW at back aperture) 200-300μm below the cortical surface to minimize the contribution of large surface vessels. A total of 12–36 trials were collected per limb, with each stimulation trial followed by a no-stimulation trial. Each 3s trial consisted of 1s of baseline followed by 1s of stimulation (or no stimulation). A piezoelectric wafer (Piezo systems, Q220-A4-203YB) was used to stimulate the contralateral forepaw or hindpaw for 1 s at 100Hz using 5ms biphasic pulses. Twelve-bit image frames (376 × 252 pixels, 16.7μm/pixel) collected all reflected red light at a frame rate of 100Hz using a MiCAM02 camera (Brain Vision). Using ImageJ software (version 1.53), the 12–36 imaging trials were averaged together and mean filtered (5 pixel radius). Stimulation induced changes in reflected light (IOS signal shown as ΔR/Ro) were calculated by subtracting and then dividing all images to an average intensity projection of pre-stimulus images (Ro). Maps of the forelimb or hindlimb somatosensory cortex were created by thresholding ΔR/Ro values at 70% of maximum intensity and superimposed on the cortical surface.

In vivo two-photon imaging and targeted delivery of AAVs to cortical regions

All mice implanted with a cranial window were allowed to recover for at least 5 weeks before experiments commenced. Mice were anesthetized with isoflurane mixed with medical air (1%) and body temperature was maintained at 37°C. Their head was fixed in place with a custom head holding apparatus. Two-photon images were collected with a 40X water immersion IR objective (Olympus, NA = 0.8) using an Olympus FV1000MPE laser scanning microscope coupled to a mode-locked Ti:sapphire femtosecond laser. For imaging Fluorescein (FITC), eGFP, eYFP, ChR2, or tdtomato/GCaMP6s, the laser was tuned to the following wavelengths: 805, 900, 920 or 945 nm, respectively. Simultaneous imaging of tdtomato and FITC dextran was achieved using 945 nm wavelength.

To visualize the cerebral vasculature and allow for subsequent puncture of targeted capillaries, we made up 0.1mL solution of 2.5–5% FITC-dextran (70kDa, Sigma-Aldrich #46945) dissolved in sterile saline solution. Various AAVs were then added to the FITC dextran solution (for a final volume of 0.1mL) and administered intravenously through tail vein or retro-orbital injection. The following AAVs were injected: 1) AAV1.hSyn.cre.WPRE.hGh (Addgene #105553; stock concentration 2.6 × 10^{13} GC/mL) at one of three doses (1.73 × 1012, 6.92 × 1012 and 1.38 × 1013 GC/kg), 2) AAV1.CAG.tdTomato (5.06 × 1012 181 GC/kg; Addgene #59462; stock concentration 1.52 × 10^{13} GC/mL), 3) AAV1.hSyn.GCamP6s.WPRE.SV40 (6.67 × 1012 182 GC/kg, Addgene #100843; stock concentration 2.5 × 10^{13} GC/mL). Before puncturing a capillary of interest, we collected a baseline image stack of the cortical vasculature at 2μm Z-steps covering an area of 317 × 317μm (1024 × 1024 pixels, 0.31μm/pixel). A flowing capillary (~3-7μm in width), 50–250μm from the pial surface was selected. Rupture was induced with our femtosecond laser, and monitored in real time with focal high power raster scanning over a region of interest (805nm, ~390 mW at back aperture, 4-5μm diameter region centered on the vessel) for 3–6 6 s. In order to maximize sampling, we typically ruptured 4–8 capillaries per mouse, spaced at least 500 μm apart from each other. To confirm AAV mediated expression of biosensors or reporter proteins, we re-imaged a volume of cortical vasculature and AAV labeled cells (~0.01–0.03 mm^3, see image stack parameters above) 2–5 weeks after capillary rupture. For GCamP6s imaging, single plane images were collected at 4 or 8Hz covering an area of 158.4 × 158.4μm. Sensory evoked calcium transients were elicited with vibrotactile stimulation of the contralateral limb at 100Hz for 1.5s, starting 5s into each 10s trial, and repeated over 6–10 trials. Sensory evoked GCaMP6s signals were calculated by extracting fluorescence values from the soma of interest. Neuropil fluorescence surrounding each cell was subtracted from the soma fluorescence to estimate ''true'' soma fluorescence. Corrected soma GCaMP6s fluorescence was then subtracted and divided by pre-stimulus soma fluorescence (Fo was defined as median F value before stimulus) to yield a percent ΔF/Fo value.

In vivo imaging of cortical dendrites in thy1-YFP-H line mice began 5 weeks after installation of the cranial window. To minimize breathing artifact motion, mice were anesthetized with an intraperitoneal injection of ketamine/xylazine (100 and 10 mg/kg, respectively) and fitted into a custom head holding frame. High resolution image stacks were acquired at 920nm with a water immersion 40X

Olympus objective (NA = 0.8). Images were sampled using a Kalman filter (average 2 frames) at 0.163μm per pixel in x-y and 1.25μm z-steps. Images of the same dendrites were acquired at 7 days intervals before and after rupture of a capillary. A median filter was applied to image stacks to reduce noise. For analysis of dendrite density, we maximally projected 7 optical sections (3 above and below the central plane of the capillary rupture). Images were binarized with the Shanbhag threshold. Using the concentric circle plugin in ImageJ, signal pixels associated with dendrites were quantified in increments of 5μm radiating from the center of the rupture, and then normalized to pre-rupture values. Spine density was quantified from image stacks in dendrites located within 40μm from the rupture site.

QUANTIFICATION AND STATISTICAL ANALYSIS

Statistical analysis of the data was conducted in Microsoft Office Excel or GraphPad Prism. Datasets were first checked for normality and outliers were identified using a ROUT test set at 1% (Figures 4B–4D). Statistical analysis of dendrite density was based on one-sample t-tests (Figure 2B; 7 ruptures from 4 female mice), whereas changes in spine density were assessed with a 1-way ANOVA (Figure 2C; 40 branches sampled in 4 female mice). For data in Figure 4B, the non-parametric Kruskal-Wallis statistic with post-hoc Dunn's test were used to analyze dose dependent differences in cell labeling per site (left panel), whereas cell type specific differences were assessed with a Mann-Whitney test (right panel; 1.73×10^{12} GC/kg: n = 24 bleed sites in 3 male mice; 6.92×10^{12} GC/kg: n = 20 bleed sites in 4 mice; 1.38×10^{13} GC/kg: n = 11 bleed sites in 3 male mice). Cell type differences in the distance to the rupture were analyzed with an unpaired t-test (Figure 4C; 1.73×10^{12} GC/kg: n = 18 bleed sites in 3 male mice). Linear regression was used to test the relationship between the extent of cell labeling and diameter of punctured capillaries (Figure 4D, n = 44 bleed sites in 7 male mice from low and medium dose group). All p values <0.05 were considered statistically significant. All the data are presented as mean ± standard error.

Cell Reports Methods, Volume *3*

Supplemental information

Optical opening of the blood-brain barrier for targeted and ultra-sparse viral infection of cells in mouse cortex

Patrick Reeson, Roobina Boghozian, Ana Paula Cota, and Craig E. Brown

Supplementary Information

Figure S1. Comparing the spatial extent of cre-mediated labelling of cells following intracortical micro-injection of AAV.hSyn.cre versus rupture of a single capillary, related to Figure 1. Confocal images from a coronal brain section show cre dependent expression of tdtomato 3 weeks after AAV injection with a glass micropipette (A; 0.4uL, ~$1.3x10^{10}$ GCs) or after rupture of a capillary (B; i.v. injection of $6.92x10^{12}$ GCs/kg). Scale bar = 200μm.

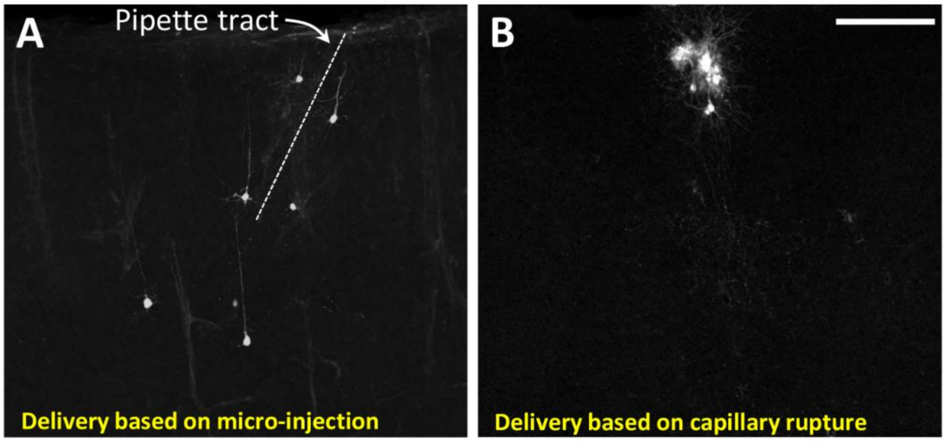

Figure S2. Time-dependent changes in microglial responses to capillary rupture, related to Figure 2 and 5. A.) Longitudinal *in vivo* imaging of microglia in male Cx3cr1$^{gfp/wt}$ mice before and up to 28 days after rupture of a single capillary. Note the rapid accumulation of microglia processes around the bleed site and delayed recruitment of cells 1 day after injury. The capillary is retained and regains blood flow while microglial reactivity subsides over 14 days after rupture. B.) Representative examples from 4 additional mice at day 0, 1, 7 and 14. Scale bars = 20μm.

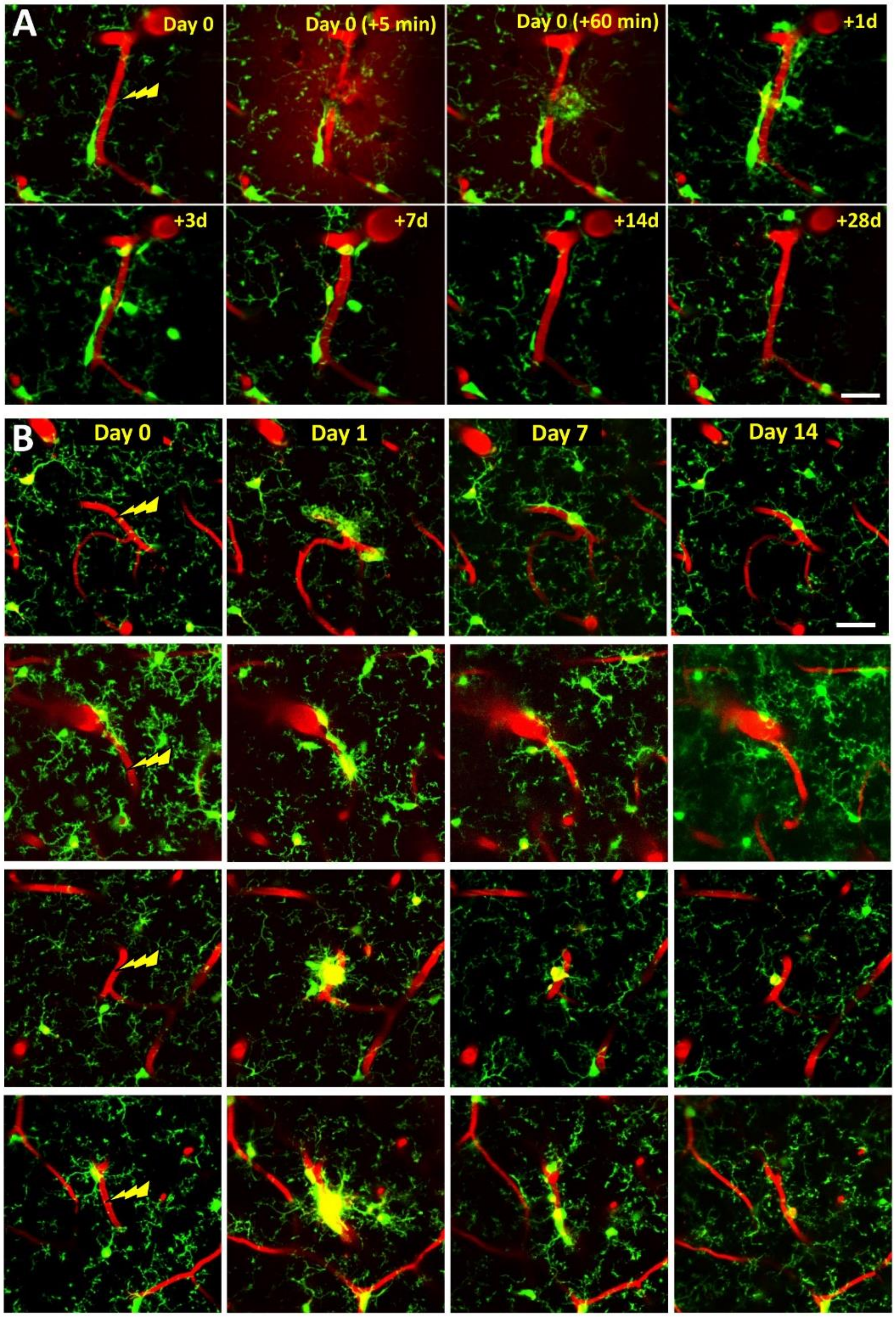

Supplement: Document S2. Article plus supplemental information [file mmc2.pdf]
